# Epiblast cells gather onto the anterior mesendoderm and initiate brain development without the direct involvement of the node in avian embryos: Insights from broad-field live imaging

**DOI:** 10.3389/fcell.2022.1019845

**Published:** 2022-10-05

**Authors:** Koya Yoshihi, Hideaki Iida, Machiko Teramoto, Yasuo Ishii, Kagayaki Kato, Hisato Kondoh

**Affiliations:** ^1^ Faculty of Life Sciences, Kyoto Sangyo University, Kyoto, Japan; ^2^ National Institute for Basic Biology, Okazaki, Japan; ^3^ Department of Biology, School of Medicine, Tokyo Women’s Medical University, Tokyo, Japan; ^4^ Exploratory Research Center on Life and Living Systems (ExCELLS), National Institutes of Natural Sciences, Okazaki, Japan; ^5^ JT Biohistory Research Hall, Takatsuki, Japan

**Keywords:** epiblast, node, anterior mesendoderm, brain primordia, chicken embryo, live imaging article type: review article

## Abstract

Live imaging of migrating and interacting cells in developing embryos has opened a new means for deciphering fundamental principles in morphogenesis and patterning, which was not possible with classic approaches of experimental embryology. In our recent study, we devised a new genetic tool to sparsely label cells with a green-fluorescent protein in the broad field of chicken embryos, enabling the analysis of cell migration during the early stages of brain development. Trajectory analysis indicated that anterior epiblast cells from a broad area gather to the head axis to form the brain primordia or brain-abutting head ectoderm. Grafting the mCherry-labeled stage (st.) 4 node in an anterior embryonic region resulted in the anterior extension of the anterior mesendoderm (AME), the precursor for the prechordal plate and anterior notochord, from the node graft at st. 5. Grafting the st. 4 node or st. 5 AME at various epiblast positions that otherwise develop into the head ectoderm caused local cell gathering to the graft-derived AME. The node was not directly associated with this local epiblast-gathering activity. The gathered anterior epiblast cells developed into secondary brain tissue consisting of consecutive brain portions, e.g., forebrain and midbrain or midbrain and hindbrain, reflecting the brain portion specificities inherent to the epiblast cells. The observations indicated the bipotentiality of all anterior epiblast cells to develop into the brain or head ectoderm. Thus, a new epiblast brain field map is proposed, allowing the reinterpretation of classical node graft data, and the role of the AME is highlighted. The new model leads to the conclusion that the node does not directly participate in brain development.

## Introduction

Many classical conceptions in developmental biology originated from the approach of experimental embryology. Typically, embryos receiving some surgical operations were examined later, e.g., the next day, to evaluate the effect of the operation on embryo histogenesis. Then, the process by which the operation affected histogenesis was inferred, and the regulation occurring in normal development was hypothesized. Such models reflected the best knowledge and considerations of the time. However, the limitations were immense due to the lack of information on the cellular events following the surgical operation. Recent technological advances in the live imaging of cellular processes have led to revolutions in understanding developmental regulation. In this review article, we discuss the unanticipated discoveries we made concerning the regulation of epiblast cell migration leading to brain tissue development.

The cell dynamics in the anterior epiblast leading to brain development have remained uninvestigated. We sparsely labeled epiblast cells in a large area with EGFP for live imaging of the broad embryonic field and analyzed the cell trajectories during brain development with or without grafts of exogenous nodes or anterior mesendoderm in the anterior embryonic field. The analysis revealed the following essential characteristics of the anterior epiblast cells. 1) Anterior epiblast cells from a broad area gather to the head axis underlain by the anterior mesendoderm (AME) during stage (st.) 5-6 and develop into brain tissues. The AME is the tissue that protrudes anteriorly from the node and penetrates into the preformed endoderm at st. 5, which subsequently develops into the axial mesodermal tissues, prechordal plate (PP), and the anterior notochord (ANC) at st. 6. 2 (see Box 1). The brain precursors occupy a square area of ∼800 μm x ∼800 µm of the epiblast surrounding the node, where the forebrain (FB), midbrain (MB), and hindbrain (HB) precursors are arranged in an anterior-to-posterior order. The more remotely located epiblast cells also gather toward the midline and develop into the head ectoderm abutting each brain portion. 3) Grafting the st. 4 node or st. 5 AME at various anterior epiblast positions that would otherwise develop into the head ectoderm resulted in the local gathering of epiblast cells onto the graft-derived AME. These cell gatherings developed into specific brain portions reflecting the developmental potential inherent to epiblast cells. The node was not directly associated with these activities. Overall, the observations indicate that the anterior epiblast cells are bipotent for brain or head ectoderm development and bear regional specificities for brain portion development.

The synthesis of the current analyses and classical observations led to the new model of the epiblast brain field and the recognition of the critical role of the AME in brain development, ruling out the direct participation of the node’s activity.

## Two initial paradoxes

**FIGURE 1:**
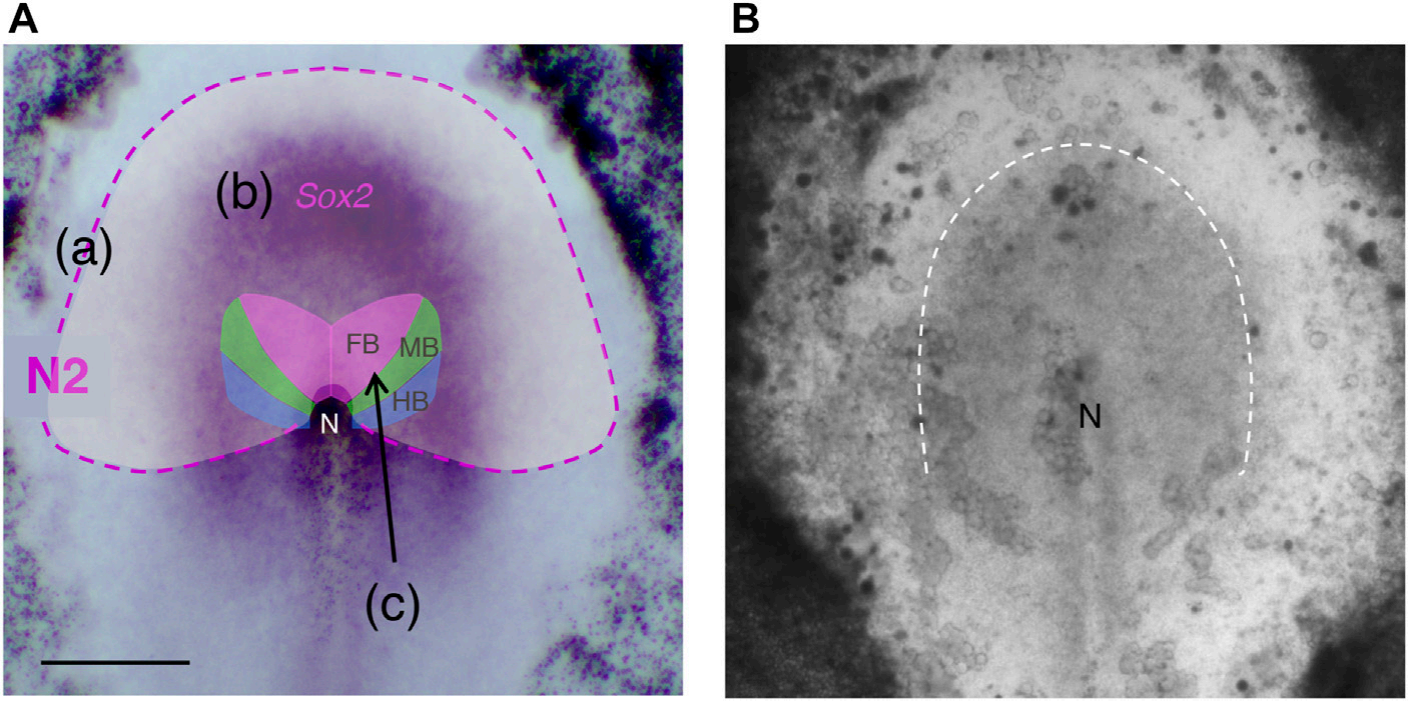
Regional characteristics of the anterior epiblast in the st. 5 chicken embryo. **(A)** Superimposition of the region with N2 enhancer activity, *Sox2* expression domain, and the map from Fernández-Garre et al. (2002) of the precursors for the brain portions, the forebrain (FB), midbrain, (MB), and hindbrain (HB). **(a)** The N2 enhancer activity region is shown in pale white with the outer lining shown as a magenta dashed line. **(b)** *Sox2* expression domain at mid st. 5 is shown by the *in situ* hybridization signal (purple). **(c)** The map by Fernández-Garre et al. (2002) drawn using homotopic grafting of labeled epiblast disks of ∼100 µm diameter is shown as a reference. The map was drawn for st. 4 embryos. However, the same map holds for early st. 5, as anterior epiblast cell migration is minimal until early st. 5. The brain precursor map was revised as shown in Figure 3 using the trajectory analysis of single-cell resolution in our study (Yoshihi et al., 2022). The anterior side is directed upward. N, the node. Scale bar, 500 µm. This panel was adapted from Yoshihi et al. (2022) Supplementary Figure S1. **(B)** A translucent image of a cultured chicken embryo of the stage analogous to **(A)**, taken from a frame of Supplementary Movie S1. A cloudy region outlined by the broken line indicates the population of epiblast cells converging to the midline.

*Sox2* gene expression, which is deeply involved in neural primordia development, is sustained via the sequential activation of a series of enhancers with distinct regional coverages (Uchikawa et al., 2003; Okamoto et al., 2015). The first turned-on *Sox2*-activating enhancer in chicken embryogenesis is N2, which covers the entire anterior half of the epiblast (Uchikawa et al., 2003; Iwafuchi-Doi et al., 2011) (Figure 1A). Despite the broad anterior coverage by this enhancer, only the narrow region of the anterior epiblast close to the midline activates the *Sox2* gene. The brain precursor region reported by Fernández-Garre et al. (2002), although revised by our study, occupied even a smaller part. We hypothesized that the broad N2-active area indicates the region with the potential to activate *Sox2* and hence to develop into neural tissue, although the potential may not be expressed in normal development.

It is generally considered that the median area pellucida of the blastoderm represents the embryogenic region, whereas the surrounding area opaca produces extraembryonic tissues (e.g., Bellairs and Osmond, 2014). However, the size and shape of the area pellucida at st. 4 vary significantly among embryos and are laterally asymmetrical (some examples shown in Figures 4, 6, 7). Nevertheless, the embryos developing from these blastoderms are almost identical in shape and size. Therefore, regional attribution of the area pellucida and area opaca to the embryonic and extraembryonic tissues, respectively, does not appear strict.

These paradoxes suggest that our current knowledge of embryogenic cellular events is lacking critical information. We started the study in the quest for the missing cellular events focusing on brain development using live imaging of epiblast cells and their interactions.

## Long-range cell migration of anterior epiblast cells to form the brain portions and covering head ectoderm

The cell migration properties in the posterior half of chicken embryos during the primitive streak-forming stage have been investigated intensively because dynamic and variably patterned cell migration occurs (Chuai et al., 2012; Rozbiki et al., 2015; Voiculescu, 2020). In contrast, epiblast migration in the anterior part of embryos, which occurs only after streak-forming stages (Rozbiki et al., 2015), has remained uninvestigated. We thus started the analysis of anterior epiblast cell migration from the st. 4 onward.

Time-lapse bright-field recordings of cultured embryos starting from st. 4 (Figure 1B and Supplementary Movie S1) indicated that a cloudy cell mass converged toward the midline before the brain tissue developed, suggesting the occurrence of the anterior epiblast cells’ massive migration. To corroborate this model and to analyze cell migration leading to brain development in detail, we devised a technique to sparsely label epiblast cells with EGFP using electroporation of the “Supernova” vector cocktail (Yoshihi et al., 2022). An additional advantage of this cell-labeling technique was that the fluorescence intensity increased only modestly over time, allowing an 18 h live recording with a constant photosensitivity setting.

**FIGURE 2:**
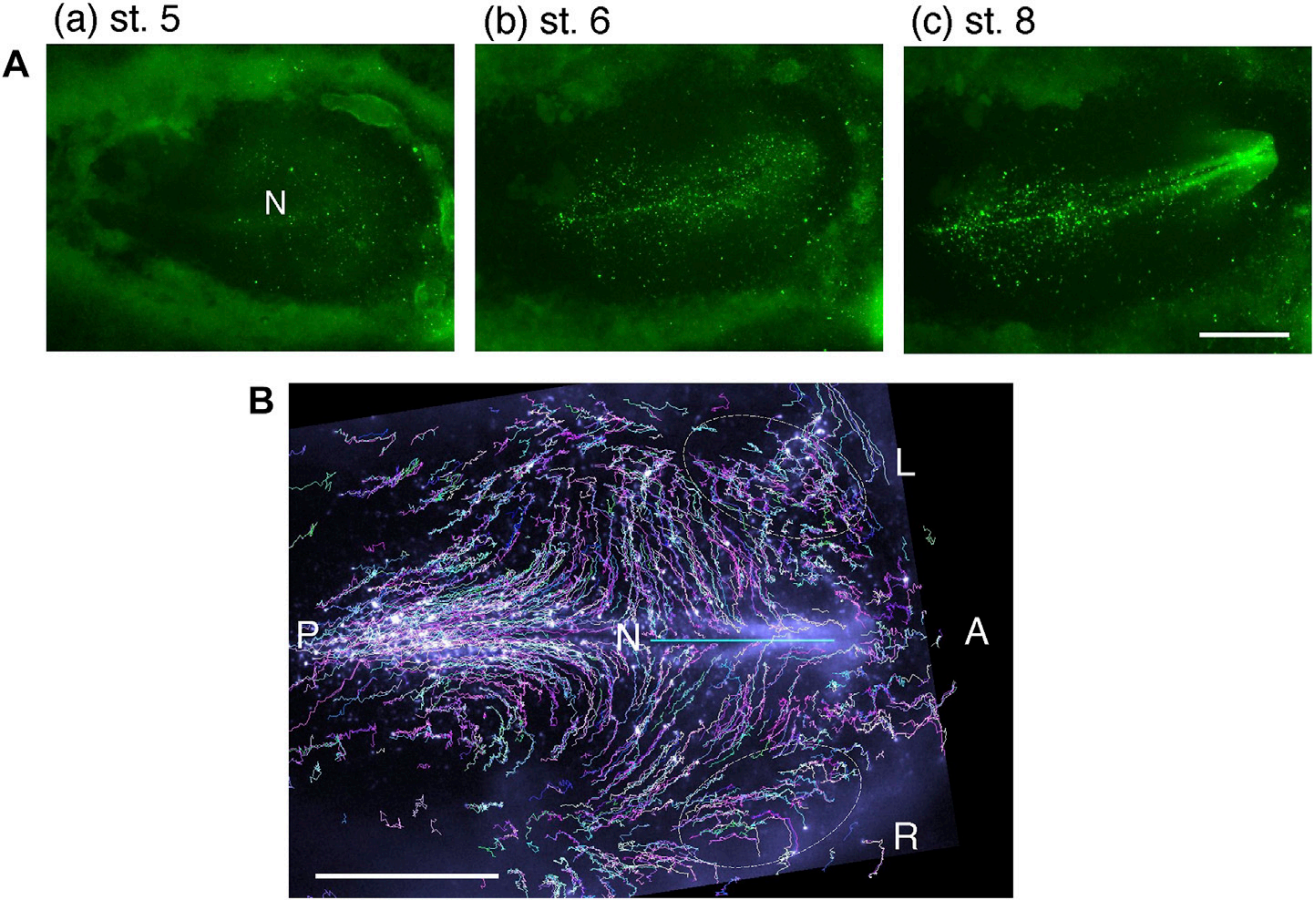
Long–range axial convergence of sparsely EGFP-labeled anterior epiblast cells. **(A)** Snapshots of an EGFP–labeled chicken embryo at different developmental stages; excerpt frames taken from Supplementary Movie S2. **(B)** Trajectories of EGFP-labeled cells were drawn in random colors to distinguish individual lines during st. 5–8 of the same embryo, taken from Supplementary Movie S3. Broken ovals indicate the cell trajectories showing cell migration across the area pellucida/area opaca boundary. Scale bars, 1 mm. Adopted from Figure 1A of Yoshihi et al. (2022).

Figure 2A and Supplementary Movie S2 represent one such recording. The sparsely labeled cells in a broad anterior epiblast region migrated long distances toward the midline. The trajectories of the labeled cells delineated the migration profiles (Figure 2B, Supplementary Movie S3). It was noted that whereas the majority of anterior epiblast cells migrated inward, many epiblast cells near the area pellucida/opaca boundary freely moved into the area opaca region (Figure 2B, enclosed by ovals). This observation indicated that the anterior epiblast cells migrate seamlessly across the area pellucida/area opaca boundary.

**FIGURE 3:**
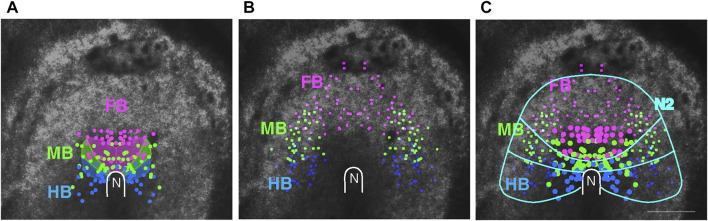
The distribution of head tissue precursors at st. 5. **(A)** Positions of the precursors for different brain portions, FB, MB, and HB, indicated by color-coded dots, compiled from data of four embryos. The data are compared with the brain portion precursor map by Fernández-Garre et al. (2002), shown as shaded areas. **(B)** The distribution of the precursors for the dorsal brain/head ectoderm at st. 8/9 in three embryos, color-coded according to the abutting brain portions. **(C)** Combination of the data in **(A,B)**, where the larger dots represent brain portion precursors. Approximate boundaries of the above precursor regions and the outer limit of *Sox2* N2 enhancer activity (Figure 1A) are drawn in cyan. N, the node position. Scale bar, 500 µm. Adopted from Figure 4 of Yoshihi et al. (2022).

The live-recorded embryos were fixed and hybridized with probes for *Otx2* (expressed in the FB and MB) and *Gbx2* (expressed in the HB) to determine the relationship between the cell migration endpoints and brain portions. By tracing back the labeled cells’ trajectories to earlier stages, a brain portion precursor map could be drawn for st. 4 to early st. 5 (when the cell migration in the anterior epiblast was minimal). The cumulative data are shown in Figure 3A, indicating that the FB, MB, and HB precursors are organized anterior-to-posterior in the ∼800 μm x ∼800 µm square region of the epiblast surrounding the node, revising the map of Fernández-Garre et al. (2002). In the scheme, the precursor distribution overlapped between the brain portions, but the overlaps reflected embryo-to-embryo variations in the A/P limits of the precursor distribution; in the data of individual embryos, their distributions did not overlap.

An interesting observation was that the precursors for the head ectoderm covering individual brain portions were distributed as a continuous extension of the same brain portions (Figure 3B,C). This observation, in combination with the hypothesis that *Sox2* N2 enhancer activity covering the entire anterior epiblast provides neural potency, as discussed above, suggested a model in which whether an anterior epiblast cell develops into brain tissue depends on whether the cell reaches the head axis-proximal zone. Those migrating from a distance have less chance of doing so and develop into the proximal head ectoderm.

BOX 1 **Glossary**

**Node:** The primitive streak involved in the genesis of trunk tissues and extending from the posterior side of a st. 2 to 3 chicken embryo stops anterior extension after approximately 17–18 h of incubation and then forms a thickened “node” tissue at its anterior end. Node formation marks the start of st. 4 (Hamburger and Hamilton, 1951). At the earliest stage of st. 4, some node cells develop into the endoderm (Yoshihi et al., 2022). After this moment, the cells around the node remain static. Then, at approximately 20 h, the node begins to extend a process anteriorly, marking the start of st. 5 (Hamburger and Hamilton, 1951). St. 5 lasts ∼3 h until the AME is fully extended, which is followed by st. 6, where the neural plate forms and the AME differentiates into the prechordal plate (PP) and anterior notochord (ANC). At the end of st. 5, the posterior notochord (PNC) originating from the st. 4 node starts to extend posteriorly, ∼3 h later than AME extension. The node tissue marked at st. 4 develops only PNC at st. 5 as the posterior component. In contrast, the node tissue marked at st. 5 also generates the median portions of the somites (Yoshihi et al., 2022), indicating that the PNC component of st. 5 node tissue is carried over from st. 4; however, the somitogenetic components are due to a new addition to the node, possibly from the neighboring epiblast. The extending posterior end of the PNC is the neurocaudal hinge, which accounts for the observation of the common origin of the notochord and the floor plate, both derived from the node (Catala et al., 1996; Teillet et al., 1998). Classical studies of chicken embryos refer to the morphological changes during this stage, such as “regression of the primitive streak with the node at its anterior end.” However, the genuine node no longer exists at this stage. In fact, the posterior expansion of the axial tissues posteriorly marked by the neurocaudal hinge replaces the primitive streak structure.

**FIGURE 4:**
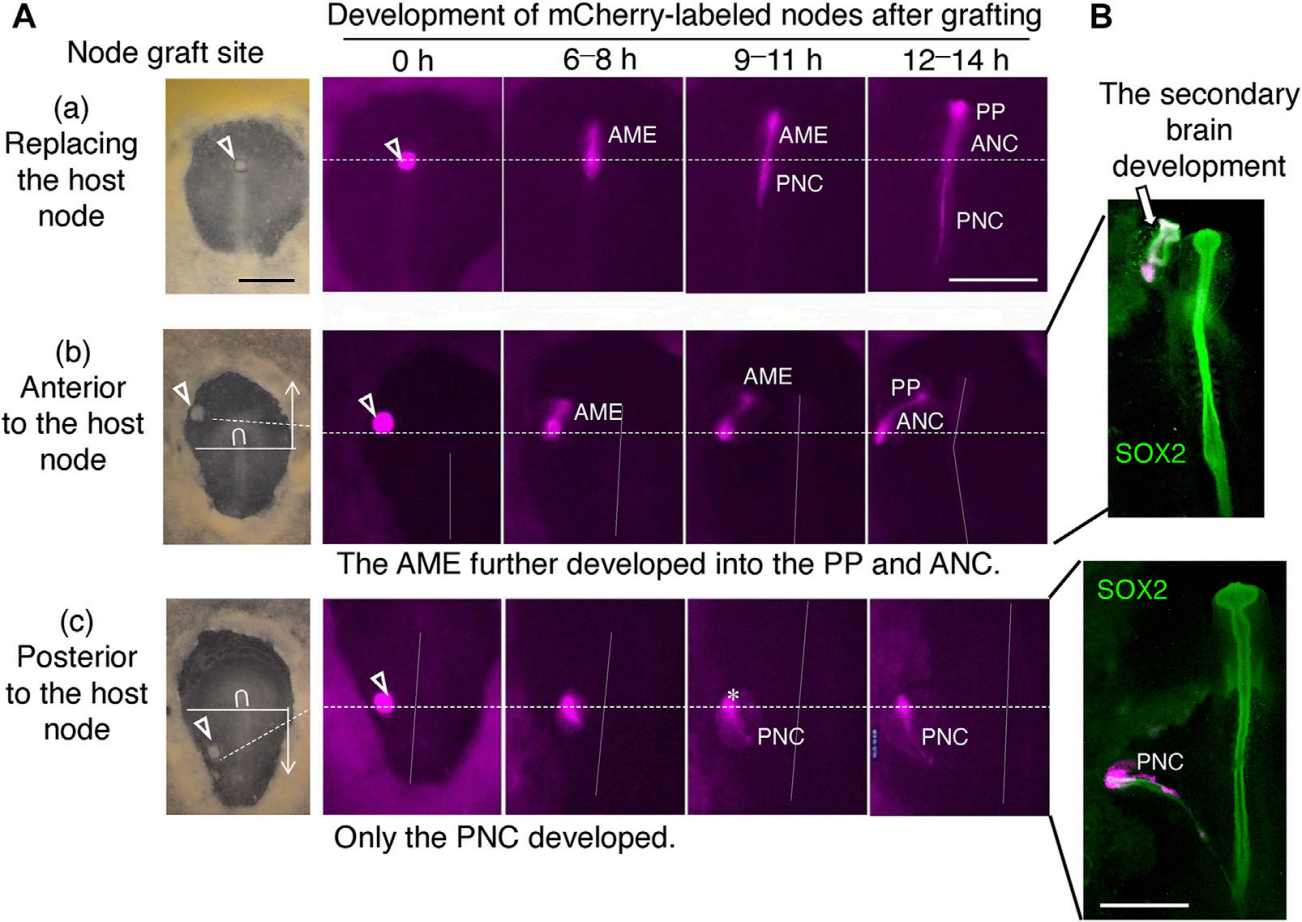
The developmental outcome of the node graft labeled with mCherry depended on the graft sites. **(A)** Development of the node grafts from mCherry-expressing st. 4 Japanese quail embryos at various positions of the chicken host embryos: **(a)** Replacing the host node; **(b)** Anterior to the host node; **(c)** Posterior to the host node. Inverted U, host node; horizontal solid lines, the boundary of anterior and posterior embryonic regions; broken horizontal lines, the anteroposterior level of the node grafting; vertical dotted lines, the host embryo axes; scale bar, 500 μm; AME, anterior mesendoderm; PP, prechordal plate; ANC, anterior notochord; PNC, posterior notochord. **(B)** Embryos analogous to **(b,c)** were immunostained for SOX2. Node grafting at an anterior position always resulted in secondary brain development expressing SOX2. Scale bar, 500 µm. Adapted from Supplementary Figure S7 of Yoshihi et al. (2022).

**Anterior mesendoderm (AME):** As shown in Figure 4A, the node extends a mesendoderm tissue anteriorly during st. 5, penetrating the premade endoderm tissue and subsequently developing into the prechordal plate (PP) and the anterior notochord (ANC). Earlier studies noted that some activities of the PP underlying the forebrain are involved in brain development, but the studies did not adequately consider the ANC portion, the structure of which continues to the posterior notochord. However, our study demonstrated that the entire length of the AME before developing into the PP and ANC displays an activity essential in gathering surrounding anterior epiblast cells and allowing them to develop into the brain tissues.

## The developmental outcome of exogenous node grafts entirely depends on whether the graft position is in the anterior or posterior half of the host embryo

To test the developmental potential of the epiblast, we set forth to graft st. 4 nodes from different embryos using the technique shown in Supplementary Movie S4. To investigate the development of the grafted node in the host embryo, nodes from transgenic Japanese quail embryos expressing mCherry (Huss et al., 2015) were used.

The developmental outcome of the grafted node in the host embryo differed greatly depending on the graft position (whether in the anterior or posterior half of the embryo) (Figure 4A). The anterior and posterior embryonic regions are separated at the axial level corresponding to the posterior limit of the brain precursors approximately 300 µm posterior to the anterior end of the node (Figure 4). The node in the original position develops into an anterior extension of the AME at the beginning of st. 5 and into a posterior extension of the posterior notochord (PNC) at the end of st. 5 (Figure 4Aa). Thus, the grafting of the mCherry-labeled node in the node position allowed the development of both anterior and posterior components. Grafting the node in an anterior embryonic position outside the node caused only the AME extension that occurred synchronously with the host AME (Figure 4Ab). Under this condition, the secondary head tissue developed (Figure 4B, upper panel), as detailed below. In contrast, when the node graft was placed at a posterior level, it developed only PNC synchronously with the host PNC (Figure 4Ac), without accompanying development of secondary trunk tissues, except for a floor plate-like tissue that lay along the PNC (Figure 4B, lower panel).

BOX 2 **A history of avian embryo culturing and node grafting**

Conrad H Waddington introduced a modern approach to the investigation of early-stage embryogenesis, namely, flat mount preparation of embryo culture (Waddington, 1932). He placed isolated avian embryos of st. 3 to 5 [according to later defined Hamburger-Hamilton (HH) stage (Hamburger and Hamilton, 1951)] freed from the yolk and oriented with the hypoblast side up on a chicken plasma clot supplemented with embryo extract. The culture method made the embryos amenable to surgical operation and observations to follow the developmental consequence of the procedure. The developmental progression of chicken embryos under Waddington’s culture was delayed in comparison to normal embryogenesis and tended to exhibit morphological abnormalities concerning tissue details. However, the technique was sufficient to meet Waddington’s aim, which was to see whether such a tissue can be found in chicken embryos, that is, analogous to the organizer reported by Hans Spemann in amphibian embryos (1924). He deemed the primitive streak of avian embryos analogous to the blastopore dorsal lip of gastrulating amphibian embryos. Waddington therefore isolated various elongated pieces of primitive streak tissue from one embryo and grafted them in isolation into the second cultured embryos at lateral positions in a space between the epiblast and hypoblast. He found some analogies between the amphibian blastopore dorsal lip and the avian primitive streak but also noted substantial differences. One of his conclusions was that only when the primitive streak graft contained the anterior end (node) did the secondary brain tissue develop (Waddington, 1932; Waddington and Schmidt, 1933). His experimental design was limited from a modern perspective. For instance, the stages of the donor and host embryos were not clearly defined. In addition, the insertion of the donor tissue in the lumen between the epiblast and hypoblast resulted in culturing of the grafted tissue without forced integration into the host tissue [analogous to the later employed “Einsteck” grafting in contrast to the original Spemann and Mangold (1924) style grafting in amphibian embryos]. Nevertheless, this study was monumental in starting an era of analytical studies into developmental biology. Waddington is often referred to as the founder of node grafting, but he never grafted a node in isolation.

In 1955, Denis New reported a new culture method for early-stage chicken embryos that allowed better development of embryos ex ovo. In the method, the embryo primordium (blastoderm) is suspended with the hypoblast side oriented upward and attached to the vitelline membrane. The vitelline membrane is lightly stretched by wrapping the cutting edge around a glass (or metallic) ring, and the embryo-vitelline membrane complex is placed on a layer of thin albumen. This culture allows normal ex ovo development of embryos up to the three-day stage of incubation, with the same pace as in ovo at least up to the 20-somite stage (New, 1955).

The classic node graft experiments pioneered by the groups of Gary Schoenwolf and Claudio Stern (e.g., Dias and Schoenwolf, 1990; Storey et al., 1992) took advantage of New’s culture technique. In these experiments, the node pieces excised from second embryos were grafted at the periphery of the area pellucida, inserted into a pouch of germinal vesicles formed at the anterior margin of the area pellucida or pushed into the inner periphery of the thickened area opaca. The choice of these embryonic sites for node grafting could have been for a technical reason. Embryos were only suspended on the vitelline membrane and tended to sway during embryo handling using New’s technique, which made the type of accurate tissue operation exemplified by Supplementary Movie S4 very challenging. These classic node graft positions traveled across the brain portion-specified domains of the epiblast, and data were reinterpreted to establish the new model (Figure 7A).

In 2001, Chapman et al. reported a revised version of the chicken embryo culture technique. The vitelline membrane holding the blastoderm was supported on filter paper with four merging punch holes and placed on the thin albumen in soft agar. We modified the procedure of Chapman et al. (2001) using a filter paper ring support, which applied uniform tension to the blastoderm in all directions (Uchikawa et al., 2003; Uchikawa et al., 2017). The use of a filter paper ring support allowed electroporation of DNA in the broad embryonic area with consistent efficiency. We realized that tissue operations on embryos, such as node grafting in the middle of the area pellucida, can be executed with high precision using our culture platform. In the study of Yoshihi et al. (2022), chicken embryo electroporation and node/AME grafting were combined using our chicken embryo culture technique.

## Node graft-derived AME or isolated AME elicits local gathering of anterior epiblast, leading to secondary brain tissue development

**FIGURE 5:**
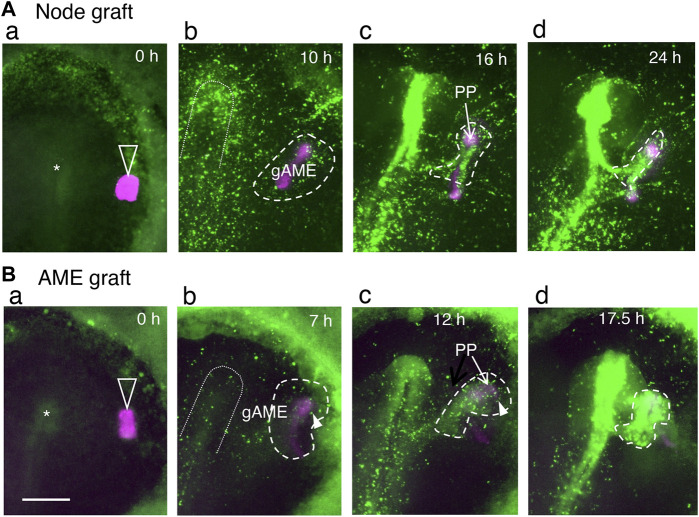
AMEs extended from the grafted st. 4 node or isolated st. 5 AME elicits the gathering of surrounding epiblast cells, which develop into secondary brain tissues, as indicated by live imaging of the fluorescent-labeled epiblast and node/AME. Adopted from **(B,D)** of Yoshihi et al. (2022). The anterior side is at the top. Open arrowheads indicate the node/AME grafts (magenta); asterisks indicate the host nodes. Broken lines encircle the epiblast cells gathering around the gAME and forming secondary brain tissues. The inverted U-shapes in dotted lines indicate the primary neural plate regions at the prospective and forming stages. **(A)** Grafting an mCherry-expressing quail node at a lateral position of the EGFP-labeled host epiblast. Excerpts from Supplementary Movie S5. **(B)** Grafting a st. 5 quail AME isolated according to Qiu et al. (1998). Excerpts from Supplementary Movie S6. The periods in the culture are indicated in panels. **(a)** The stage when the quail node/AME was grafted. **(b)** The graft-derived AME (gAME) elongated and elicited the gathering of nearby epiblast cells (encircled by broken lines). **(c)** The gAME tissue differentiated into the PP (indicated by an arrow) and ANC (covered by EGFP fluorescence). The head precursors that converged on gAME started to form secondary brain tissues (encircled by broken lines). **(d)** The secondary brain (encircled by broken lines) fused to the primary brain at the posterior end in these specimens. Arrowheads in **(b,c)** indicate the contribution of cells derived from area opaca to the secondary brain tissue. Scale bar, 500 µm.

As the grafting of nodes in the anterior part of embryos always causes secondary brain development, the sequence of events following the node grafting was investigated in detail using host embryos with Supernova-labeled epiblast cells and mCherry-labeled node grafts from transgenic quails (Huss et al., 2015). On all occasions of anterior node grafting, the grafted node started to extend the AME at st. 5, then the surrounding epiblast cells responded to the AME and gathered around it. The gathering of epiblast cells developed into secondary brain tissue, which in most cases fused to the host brain at the posterior end (Figure 5A; Supplementary Movie S5). The anterior epiblast cells did not gather at the node. As shown in Supplementary Movie S5, the labeled epiblast cells initially positioned next to the node migrated away, but as the AME started to extend, the surrounding labeled cells gathered around the AME.

mCherry-labeled st. 5 AME freed from the node tissue was grafted underneath the st. 4 host epiblast cells labeled with EGFP to confirm that the AME without node involvement allowed proximal epiblast cells to gather and allowed the cell gathering to develop into brain tissue. After all such AME grafting, the host epiblast cells quickly gathered around the AME graft and developed into the secondary brain tissues (Figure 5B, Supplementary Movie S6). This observation confirmed that the AME promotes accumulation of proximal epiblast cells prior to their development into brain tissue and indicated that the st. 4 epiblast cells are prepared to respond to the AME once they are placed nearby.

## The development of brain portions in the secondary brain depends on the positions of the grafted AMEs

**FIGURE 6:**
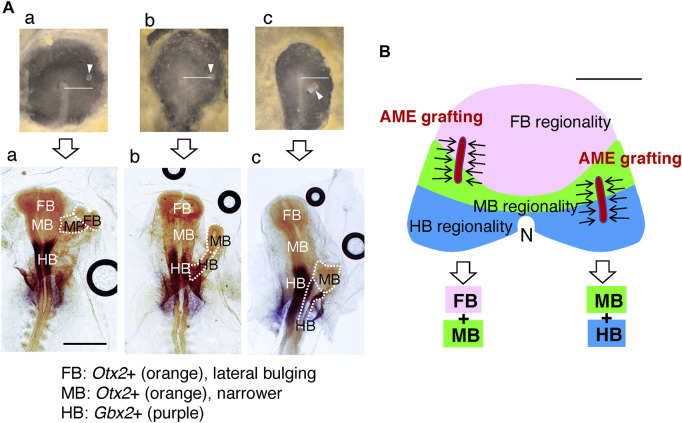
The brain portions that developed in the secondary brain depended on the AME graft positions. **(A)** Representative AME grafts at different AP levels in host embryos immediately after grafting (upper) and after ∼18 h with hybridization for *Otx2* and *Gbx2* (lower). The AME extended anteriorly from the graft site to a length of ∼500 µM. **(a)** An example of an anterior AME graft resulting in FB and MB development in the secondary brain. **(b)** The AME graft at the node level resulted in MB and HB portions in the secondary brain. **(c)** Posterior AME grafting elicited the development of inferior MB and large HB portions. In all cases, the posterior end of the secondary brain fused to the host brain at the level of the same portion, supporting the model that host and secondary brain portions develop using the pool of anterior epiblast cells of the same brain portion specificity. The horizontal bars in the upper panels extend 1 mm from the node center. Scale bar for the lower panels, 1 mm. Modified from Figure 6 of Yoshihi et al. (2022). **(B)** Upper: Divisions of the anterior epiblast field with distinct brain portion specificities, drawn according to the data in Figure 3C, and the schematic representation of the extended AME grafts gathering the proximal epiblast cells (arrows) from different divisions. Lower: The resultant composition of the brain portions in the secondary brain. These relationships were confirmed using 13 AME-grafted embryos. Two additional FB-only examples are shown in Figure 7. The host brain on the midline develops with all three FB, MB, and HB portions, because the midline AME passes through all three epiblast divisions. Scale bar, 500 µm.

Judged from the morphology, the brain portions that developed in the secondary head depended on the positioning of the exogenous AME in the anterior host field. The embryos were fixed and hybridized with the *Otx2* and *Gbx2* probes to determine the brain portions in the secondary head. As shown in Figure 6A, AME grafting at an anterior position in the anterior epiblast resulted in FB-containing secondary head development. In contrast, AME grafting at a posterior position in the anterior epiblast resulted in HB-containing secondary head development. In many embryos (*n* = 15), the AME graft positioning and the resultant brain portions formed in the secondary brain were strongly correlated. The brain portion composition in the secondary head reflected which regions of the anterior epiblast the AME passed through after full elongation of the AME to ∼500 µm (Figure 6B), the regions of the head ectoderm precursors extending from the respective brain portion precursors, as shown in Figure 3C. These observations indicated first that precursors for a brain portion and abutting head ectoderm in normal development share the same potential to develop into the corresponding brain portion. Namely, the anterior epiblast cells are initially bipotential for developing the brain tissue or head ectoderm. Second, the AME-gathered epiblast cells develop into specific brain portions, fully reflecting their inherent specificity (Figure 6B); thus, the AME does not specify which portions to develop in the secondary brain.

## Epiblast brain field

**FIGURE 7:**
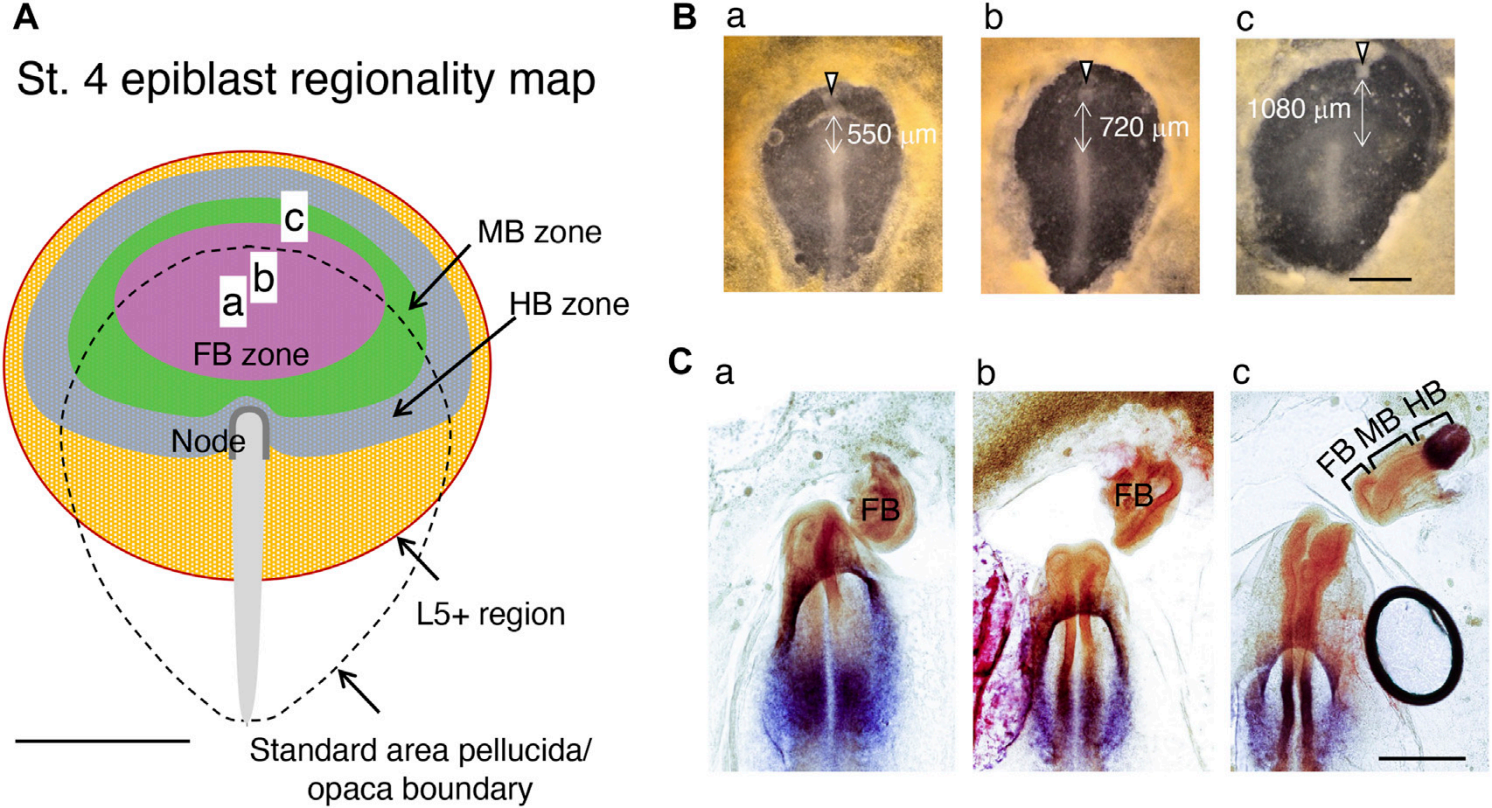
Regionality map of the st. 4 epiblasts constructed using available data. **(A)** Distribution of the head tissue developmental potential of anterior epiblast cells at st. 4. The developmental potential extends by crossing the area pellucida boundary to the L5+ domain of the area opaca (Streit et al., 1997). The zones with developmental potential for the individual brain portion are drawn using pale colors. **(a–c)** The AME graft positions in **(B)**. **(B)** Embryos with AME grafts close to the area pellucida anterior limit (arrowheads) with variable distances from the node. **(C)** The secondary brain tissues in embryos in **(B)** after 18 h, hybridized for *Otx2* and *Gbx2* expression and assessed for the brain portions. Scale bars, 1 mm in **(A,B)**, 500 µm in **(C)**. Adapted from Figure 7 of Yoshihi et al. (2022).

Then, how would the epiblast brain field with the brain portion regionality extend further? As described in the article in Box 2, classical node graft experiments were performed by placing the exogenous node at the periphery of the area pellucida. In these experiments, Streit et al. (1997) made an interesting observation that the L5 antigen is expressed widely in the orbicular region of the anterior epiblast, even covering a region of area opaca. The authors observed that the epiblast cells’ response to the node graft giving rise to a secondary brain tissue occurred only at a position with L5 expression, suggesting that L5 expression marks the epiblast brain (potential) field. When the nodes were grafted at the anterolateral aspect of the area pellucida/opaca boundary, MB and HB portions developed (Storey et al., 1992), indicating that the brain regionality map shown in Figure 3C can be extended to account for these limits of the MB and HB. In contrast, when the nodes were grafted around the anterior margin of the area pellucida, the secondary brain developed not only to include the FB portion but also the MB and often HB portions in an orientation opposite to that of the primary host head (Dias and Schoenwolf, 1990; Knoetgen et al., 2000), indicating that MB and HB regionality domains exist more anterior to the FB domain. Combining our observations in Figure 6C and the classic node graft data discussed above, we modeled a concentric brain regionality map in the L5+ epiblast brain field, as shown in Figure 7A.

To test this model, we grafted exogenous AMEs at anterior positions, either in the prospective FB-potential region or at a far-anterior area presumably encompassing all prospective FB-, MB-, and HB-potential regions (Figure 7B). The latter AME grafting utilized an embryo having a large anterior extension of the area pellucida. Hybridization of the secondary brain tissues with the *Otx2* and *Gbx2* probes, combined with a morphological criterion, indicated that grafting AMEs confined to the prospective FB region resulted in the development of FB-only secondary brains, whereas AME grafting at a far anterior position resulted in a secondary brain with a complete composition of FB, MB, and HB in an orientation opposite to that of the host head, exactly as predicted (Figure 7C). Thus, the brain field map shown in Figure 7A has been supported by all available data.

## The node is not an organizer

**FIGURE 8:**
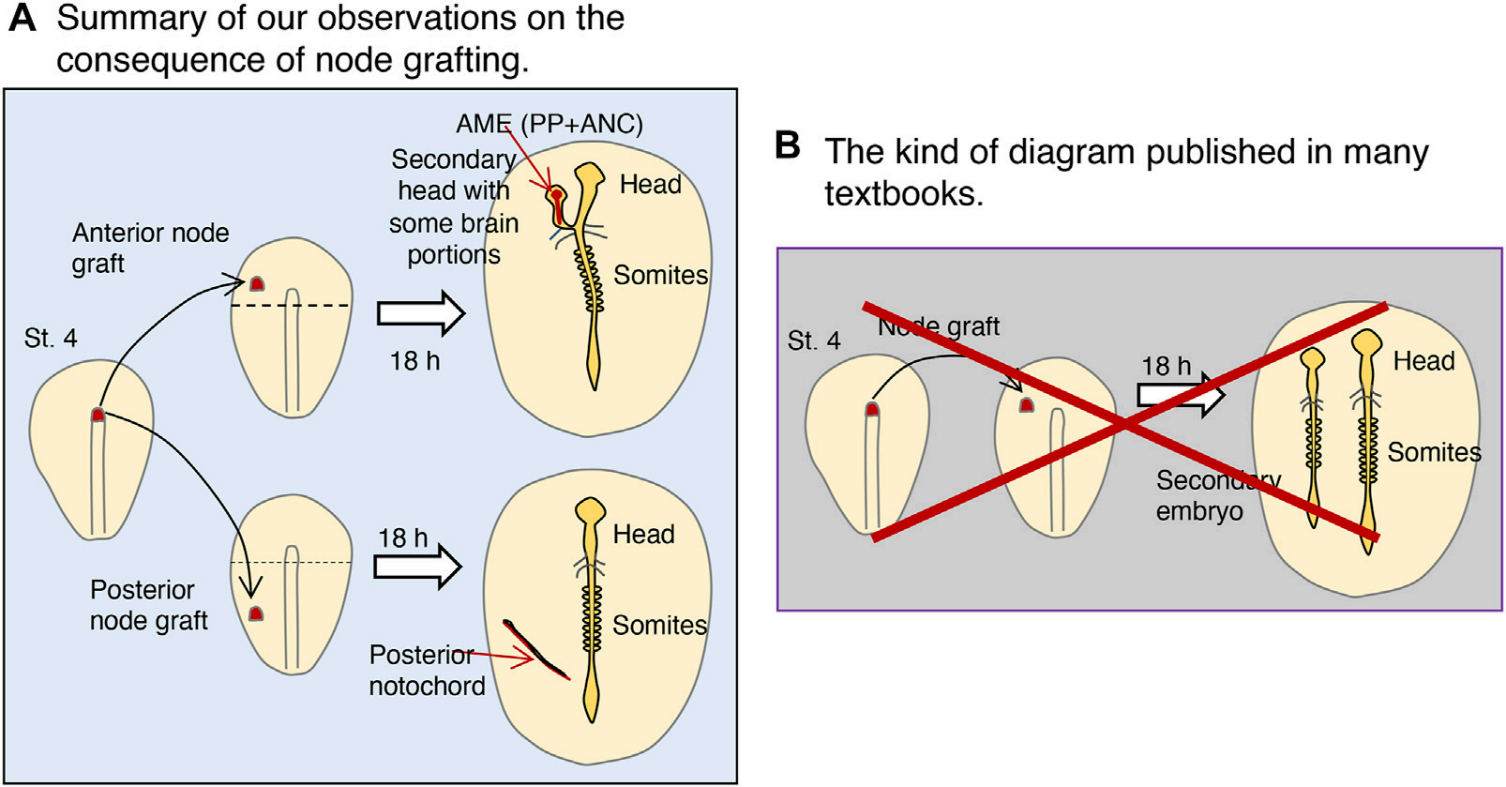
The consequence of st. 4 node grafting into st. 4 host embryos: the true and the erroneous. **(A)** Summary of our observations on the result of node grafting. The grafted node developed differently depending on whether the graft was on the anterior or posterior half of the embryo, separated by the horizontal broken line. Only after node grafting at an anterior position did the node-derived AME extend, to which the nearby epiblast converged and developed into the brain portions. **(B)** The kind of diagram published in many textbooks, which are erroneous. This method of systematic grafting has not been done previously (see Box 2). Node grafting does not elicit secondary posterior embryonic structures of host origin (e.g., somites) (see Figure 4).

Figure 8A summarizes the outcome of st. 4 node grafting in the anterior or posterior half of embryos. Anterior node grafting causes graft-derived AME development, in which proximal epiblast cells gather and develop into the secondary head structure. In contrast, posterior node grafting results in the self-differentiation of the node-derived PNC. Many textbooks include a diagram analogous to that in Figure 8B, which claims that a node graft causes the development of a secondary embryo possessing full head-to-trunk structures. However, as discussed in, no such experiments have been performed systematically before us, nor are such results expected to be possible. These diagrams were drawn with the assumption that the node acts as an organizer, as theorized by Spemann and Mangold (1924), and due to the belief that the node as an organizer must induce the entire secondary embryo structures, without any experimental verification.

If we define an organizer as the tissue that, upon ectopic grafting, gives rise to more-or-less fully equipped secondary embryos of host origin, at least the following three tissues must colocalize coincidentally in the case of the chicken embryo: 1) the st. 3 primitive streak (14–17 h), which derives mesodermal tissues; 2) the st. 4−node (17 h), which derives the endoderm; and 3) the st. 5 AME (20–23 h) on which the brain and head ectoderm develop. Even under such a hypothetical setting, tissue grafting cannot give rise to a host-derived secondary spinal cord. In any case, no single tissue exists that deserves to be called “an organizer” in avian embryos. Martinez Arias and Ben Steventon (2018), who compared mouse and amphibian embryogenesis, also concluded, “Whereas the amphibian organizer is a contingent collection of elements, each performing a specific function, the elements of organizers in other species are dispersed in time and space.” Indeed, the studies on amphibian embryos conducted by Johannes Holtfreter and his associates during the 1930s and later (reviewed in Holtfreter and Hamburger, 1955; Hamburger, 1988; Holtfreter, 1991) indicated that the dorsal lip of a blastopore is a mosaic of tissues with different developmental and interaction potentials, aligning with the current conclusions.

## Conclusion and future perspectives

The study discussed in this article highlights the power of live imaging of embryonic development, which facilitates studies not possible with the classic approach of experimental embryology. By differentially labeling the broad area of epiblast cells and the node-derived AME and analyzing their live images over 18 h, we identified previously undescribed mechanisms central to brain development: 1) AME-directed gathering of proximal anterior epiblast cells leads to the formation of the brain primordium and 2) inherent brain portion regionality is manifested following epiblast gathering.

These live imaging-based analyses of cellular behaviors provide the basis for subsequent step analyses of molecular mechanisms. For instance, what are the signals emanating from the AME that elicit the gathering of proximal epiblast cells? To address this question, we tested cocktails of some candidate signaling molecules, LEFTY1, CERL1 (both nodal antagonists), DKK1 (Wnt antagonist), and NOGGIN (BMP antagonist). However, none of their combinations mimicked the AME action in eliciting epiblast gathering or promoting brain tissue development (Yoshihi et al., 2022). The identification of signaling factors responsible for AME-dependent regulation will be crucial for elucidating the molecular processes leading to brain tissue development.

What makes the anterior and posterior epiblast so different in developmental potential? What is the basis for the regional specification of the anterior epiblast, i.e., the developmental potential for FB, MB, or HB present before the cell gathering starts? Many critical questions concerning the regulation of epiblast development have been opened due to the new perspectives provided by the live imaging of embryonic cells and their interactions.

## References

[B1] Bellairs R. Osmond M. (2014). The atlas of chick development. Third Edition. Oxford: Academic Press.

[B2] Catala M. Teillet M. A. De Robertis E. M. Le Douarin M. L. (1996). A spinal cord fate map in the avian embryo: While regressing, hensen's node lays down the notochord and floor plate thus joining the spinal cord lateral walls. Development 122, 2599–2610. 10.1242/dev.122.9.2599 8787735

[B3] Chapman S. C. Collignon J. Schoenwolf G. C. Lumsden A. (2001). Improved method for chick whole-embryo culture using a filter paper carrier. Dev. Dyn. 220, 284–289. 10.1002/1097-0177(20010301)220:3<284:AID-DVDY1102>3.0.CO;2-5 11241836

[B4] Chuai M. Hughes D. Weijer C. J. (2012). Collective epithelial and mesenchymal cell migration during gastrulation. Curr. Genomics 13, 267–277. 10.2174/138920212800793357 23204916 PMC3394114

[B5] Dias M. S. Schoenwolf G. C. (1990). formation of ectopic neurepithelium in chick blastoderms: Age-related capacities for induction and self-differentiation following transplantation of quail hensen's nodes. Anat. Rec. 228, 437–448. 10.1002/ar.1092280410 2285160

[B6] Fernández-Garre P. Rodriguez-Gallardo L. Gallego-Diaz V. Alvarez I. S. Puelles L. (2002). Fate map of the chicken neural plate at stage 4. Development 129, 2807–2822. 10.1242/dev.129.12.2807 12050131

[B7] Gomez C. Ozbudak E. M. Wunderlich J. Baumann D. Lewis J. Pourquié O. (2008). Control of segment number in vertebrate embryos. Nature 454, 335–339. 10.1038/nature07020 18563087

[B8] Hamburger V. Hamilton H. L. (1951). A series of normal stages in the development of the chick embryo. J. Morphol. 88, 49–92. 10.1002/jmor.1050880104 24539719

[B9] Hamburger V. (1988). The heritage of experimental embryology: Hans Spemann and the organizer. Oxford: Oxford University Press.

[B10] Holtfreter J. Hamburger V. (1955). “Amphibians,” in Analysis of development. Editors Willier B. H. Weiss P. Hamburger V. (Philadelphia and London: W.B. Saunders Co), 230–296.

[B11] Holtfreter J. (1991). “Reminiscences on the Life and work of Johannes holtfreter,” in A conceptual history of modern embryology. Developmental biology. Editor Gilbert S. F. (Boston: Springer), 7. Chapter 6. 10.1007/978-1-4615-6823-0_6 1804210

[B12] Huss D. Benazeraf B. Wallingford A. Filla M. Yang J. Fraser S. E. (2015). A transgenic quail model that enables dynamic imaging of amniote embryogenesis. Development 142, 2850–2859. 10.1242/dev.121392 26209648 PMC4550965

[B13] Iwafuchi-Doi M. Yoshida Y. Onichtchouk D. Leichsenring M. Driever W. Takemoto T. (2011). The Pou5f1/Pou3f-dependent but SoxB-independent regulation of conserved enhancer N2 initiates Sox2 expression during epiblast to neural plate stages in vertebrates. Dev. Biol. 352, 354–366. 10.1016/j.ydbio.2010.12.027 21185279

[B14] Knoetgen H. Teichmann U. Wittler L. Viebahn C. Kessel M. (2000). Anterior neural induction by nodes from rabbits and mice. Dev. Biol. 225, 370–380. 10.1006/dbio.2000.9834 10985856

[B15] Martinez Arias A. Steventon B. (2018). On the nature and function of organizers. Development 145, dev159525. 10.1242/dev.159525 29523654 PMC5868996

[B16] New D. A. T. (1955). A new technique for the cultivation of the chick embryo *in vitro* . Development 3, 326–331. 10.1242/dev.3.4.326

[B17] Okamoto R. Uchikawa M. Kondoh H. (2015). Sixteen additional enhancers associated with the chicken Sox2 locus outside the central 50-kb region. Dev. Growth Differ. 57 (1), 24–39. 10.1111/dgd.12185 25431100

[B18] Qiu M. Shimamura K. Sussel L. Chen S. Rubenstein J. L. (1998). Control of anteroposterior and dorsoventral domains of Nkx-6.1 gene expression relative to other Nkx genes during vertebrate CNS development. Mech. Dev. 72, 77–88. 10.1016/s0925-4773(98)00018-5 9533954

[B19] Rozbicki E. Chuai M. Karjalainen A. I. Song F. Sang H. M. Martin R. (2015). Myosin-II-mediated cell shape changes and cell intercalation contribute to primitive streak formation. Nat. Cell Biol. 17, 397–408. 10.1038/ncb3138 25812521 PMC4886837

[B20] Spemann H. Mangold H. (1924). Über induktion von Embryonalanlagen durch I implantation artfremder organisatoren. Arch. F. Mikr. Anat. U. Entwicklungsmechanik 100, 599–638. 10.1007/bf02108133

[B21] Storey K. G. Crossley J. M. De Robertis E. M. Norris W. E. Stern C. D. (1992). Neural induction and regionalisation in the chick embryo. Development 114, 729–741. 10.1242/dev.114.3.729 1618139

[B22] Streit A. Sockanathan S. Perez L. Rex M. Scotting P. J. Sharpe P. T. (1997). Preventing the loss of competence for neural induction: HGF/SF, L5 and sox-2. Development 124, 1191–1202. 10.1242/dev.124.6.1191 9102306

[B23] Teillet M. A. Lapointe F. Le Douarin N. M. (1998). The relationships between notochord and floor plate in vertebrate development revisited. Proc. Natl. Acad. Sci. U. S. A. 95, 11733–11738. 10.1073/pnas.95.20.11733 9751734 PMC21709

[B24] Uchikawa M. Ishida Y. Takemoto T. Kamachi Y. Kondoh H. (2003). Functional analysis of chicken Sox2 enhancers highlights an array of diverse regulatory elements that are conserved in mammals. Dev. Cell 4, 509–519. 10.1016/s1534-5807(03)00088-1 12689590

[B25] Uchikawa M. Nishimura N. Iwafuchi-Doi M. Kondoh H. (2017). Enhancer analyses using chicken embryo electroporation. Methods Mol. Biol. 1650, 191–202. 10.1007/978-1-4939-7216-6_12 28809022

[B26] Voiculescu O. (2020). Movements of chick gastrulation. Curr. Top. Dev. Biol. 136, 409–428. 10.1016/bs.ctdb.2019.11.015 31959297

[B27] Waddington C. H. (1932). Experiments on the development of chick and duck embryos, cultivated *in vitro* . Phil Trans. R. Soc. Lond B 221, 179–230.

[B28] Waddington C. H. Schmidt G. A. (1933). Induction by heteroplastic grafts of the primitive streak in birds. Wilhelm Roux Arch. Entwickl. Mech. Org. 128, 522–563. 10.1007/BF00649863 28353960

[B29] Yoshihi K. Kato K. Iida H. Teramoto M. Kawamura A. Watanabe Y. (2022). Live imaging of avian epiblast and anterior mesendoderm grafting reveals the complexity of cell dynamics during early brain development. Development 149 (6), dev199999. 10.1242/dev.199999 35132990 PMC9017232

