# Supplementary material for: Epiblast cells gather onto the anterior mesendoderm and initiate brain development without the direct involvement of the node in avian embryos: Insights from broad-field live imaging
Source: Front Cell Dev Biol. 2022 Oct 5;10:1019845. doi: 10.3389/fcell.2022.1019845 (PMC9581324; doi:10.3389/fcell.2022.1019845)
Supplement: Supplementary file 7 [file DataSheet1.docx]

Movies for

Epiblast cells gather onto the anterior mesendoderm and initiate brain development without the direct involvement of the node in avian embryos: Insights from broad-field live imaging

**by**

Koya Yoshihi, Hideaki Iida, Machiko Teramoto, Yasuo Ishii, Kagayaki Kato, and Hisato Kondoh

**Movie legends**

**Movie 1. A movie representation of the morphological changes of embryos during brain and head formation.** Translucent images of the cultured chicken embryo were taken from the epiblast side using transmitting light illumination. The frames were taken at 25 min intervals starting from st. 4, and displayed as the movie at 6 frames per second. The primary interest of this movie is the convergence of the anterior epiblast cells starting in st. 5 [see Fig. 1(B)]. An additional interest not relevant to the issue of this review article is the initiation of somitogenesis. The first three somites were not cleaved in sequence but simultaneously. Moreover, this and analogous movies (N=6) indicated that cleavages of the 4th to 7th somites occur at ~1 hr intervals, in contrast to the 1.5 hr intervals reported for later developmental stages (Gomez et al., 2008).

**Movie 2. A representative time-lapse recording from st. 5 to st. 9 of EGFP-labeled epiblast cells using Supernova vector electroporation.** The movie visualizes the convergence of the epiblast cells toward the midline even over long distances. The frames were taken at 10 min intervals and converted to the movie at 6 fps. This movie provided the original data presented in Fig. 2(A). Adopted from Movie 1 of Yoshihi et al. (2022).

**Movie 3. Trajectories of the EGFP-labeled cells relative to the axis of the node-derived AME (magenta line) during st. 5–8.** The embryo data shown in Movie 2 were processed so that trajectories covering more than 8 consecutive frames were displayed with random coloring to distinguish individual lines. This movie demonstrates the epiblast convergence toward the AME position. The original data for Fig. 2(B). Adopted from Movie 2 of Yoshihi et al. (2022).

**Movie 4. The method of tissue handling to graft a node isolated from a st. 4 quail embryo onto an anterolateral position of the chicken embryo.** Frames are presented at real-time speed with the omission of some sequences; the handling procedure is completed in 4**–**5 minutes by skilled hands. The first half shows the excision, using a tungsten needle, of a node from a quail embryo supported in the hole of a filter paper disk laid on the thin albumen-soft agar medium. The embryo was oriented with the hypoblast facing the surface. The node, carved out as a square tissue block, was suspended in a droplet held in forceps and transferred to the host embryo graft site. A size-adjusted square hole was carefully carved from the hypoblast side, and the node block was inserted.

**Movie 5. A representative time-lapse recording of an EGFP-labeled epiblast from st. 4 to st. 9 with a grafted mCherry-labeled st. 4 Japanese quail node at a position lateral to the host node.** This movie shows the development of a node graft into a graft-derived AME (gAME) and the convergence of the surrounding epiblast cells toward the AME, which elicited secondary brain tissue development, followed by its fusion to the host brain at the posterior end. The frames were captured at 25 min intervals and shown at 6 fps. This movie provided the original fluorescent data presented in Fig. 5(A). Adopted from Movie 6 of Yoshihi et al. (2022).

**Movie 6. A time-lapse recording of an EGFP-labeled epiblast from st. 4 to st. 9 analogous to Movie 5 but with an mCherry-labeled st. 5 AME graft lateral to the host node.** The AME graft developed into the anterior-most prechordal plate (PP) and, more posteriorly, the anterior notochord (ANC). The surrounding epiblast cells gathered around the grafted AME and formed the secondary brain tissue, which was fused to the host brain at its posterior end. Note also the contribution of area opaca-derived cells in the secondary brain. The frames were captured at 10 min intervals and shown at 6 fps. This movie provided the original fluorescence data presented in Fig. 5(B). Adopted from Movie 8 of Yoshihi et al. (2022).
